# Response to letter regarding “Long-term implant survival after debridement, antibiotics and implant retention (DAIR) for acute prosthetic joint infections: is it a viable option beyond four weeks after index arthroplasty?”

**DOI:** 10.1007/s00264-025-06584-3

**Published:** 2025-07-10

**Authors:** Ernesto Muñoz-Mahamud, Andrés Combalia

**Affiliations:** 1 Orthopaedic Surgery Department, Hospital Clínic de Barcelona, Barcelona, Spain; 2 Departament de Cirurgia i Especialitats, University of Barcelona, Barcelona, Spain

We would like to thank Drs. Chen and Hsu for their interest in our article [1] and for the thoughtful comments provided in their letter [2]. We appreciate their careful reading and are pleased that our work has contributed to the ongoing discussion regarding the optimal management of acute prosthetic joint infections (PJI).

With regard to Table 1, we acknowledge that the labels “Primary THA” and “Revision THA” may have caused confusion. These terms were used to broadly describe hip or knee arthroplasties, performed either as primary or revision procedures. While we agree the terminology could have been clearer, we emphasize that the numerical data are accurate and consistent throughout the manuscript.

Concerning the request for more procedural detail, we understand the relevance of this point. Our institution follows a standard protocol whereby empirical intravenous antibiotics are administered immediately before the surgical incision—except in patients who are clinically unstable and require earlier initiation. Targeted therapy is introduced once culture results are available. Although empirical regimens evolved during the study period based on microbiological surveillance, the overarching strategy remained consistent. Likewise, modular components are exchanged whenever feasible, in line with protocol, though we acknowledge that the retrospective nature of the study did not allow us to confirm this detail in every case.

The difference in culture-negative cases between groups is indeed noteworthy. However, we would like to underline that our study was not designed to explore outcomes according to microbiological findings. Our focus was specifically on timing of DAIR after index arthroplasty. We agree that future research on microbiology-stratified outcomes is necessary.

Regarding the inclusion of patients who died with an infection-free prosthesis, this was a deliberate methodological decision. Our objective was to assess implant survival in the broadest clinical sense, and we believe these cases should not be excluded, particularly as most deaths were unrelated to the infection.

Finally, we appreciate the suggestion to consider time from symptom onset. Unfortunately, this variable could not be reliably extracted from our records, particularly in patients referred from external centres. Nevertheless, we agree it would be an important parameter in prospective designs.

Once again, we are grateful to Drs. Chen and Hsu for their constructive input, which has given us the opportunity to clarify key points. We hope this exchange contributes to advancing knowledge and practice in this challenging area.

## Data Availability

No datasets were generated or analysed during the current study.
